# Distinct Lipid Transfer Proteins display different IgE‐binding activities that are affected by fatty acid binding

**DOI:** 10.1111/all.13682

**Published:** 2018-12-16

**Authors:** Roberta Aina, Pawel Dubiela, Sabine Geiselhart, Merima Bublin, Maurizio Bruschi, Christian Radauer, Christoph Nagl, Piotr Humeniuk, Riccardo Asero, Charlotte Gotthard Mortz, Christine Hafner, Karin Hoffmann‐Sommergruber, Tomasz Borowski

**Affiliations:** ^1^ Department of Pathophysiology and Allergy Research Medical University of Vienna Vienna Austria; ^2^ Department of Earth and Environmental Science University of Milano Bicocca Milan Italy; ^3^ Ambulatorio di Allergologia Clinica San Carlo Paderno Dugnano (MI) Italy; ^4^ Department of Dermatology and Allergy Center Odense Research Center for Anaphylaxis (ORCA) Odense University Hospital Odense Denmark; ^5^ Department of Dermatology University Hospital St. Poelten Karl Landsteiner University of Health Sciences St. Pölten Austria; ^6^ Karl Landsteiner Institute of Dermatological Research Karl Landsteiner Gesellschaft St. Pölten Austria; ^7^ Jerzy Haber Institute of Catalysis and Surface Chemistry Polish Academy of Sciences Krakow Poland

**Keywords:** fatty acid binding, food allergens, IgE epitope, nonspecific lipid transfer proteins, protein structure

## CONFLICT OF INTEREST

RAi has received funding from the Marie‐Curie project CARAMEL within the 7th European Community Framework Programme. KHS, PD, SG, and PH have been supported by the Austrian Science Fund (FWF). The remaining authors report no conflicts of interest.

To the Editor,

Nonspecific lipid transfer proteins (nsLTPs) are relevant food allergens. They have a compact 3D structure, with a hydrophobic lipid‐binding cavity.^1^ It seems that the complex with a ligand can affect protein allergenicity.^2^ Recently, we have shown that the binding of oleic acid (OLE) to the peach nsLTP, Pru p 3, affects the conformation and IgE‐binding activity of the allergen.^3^ We therefore investigated whether this observation can be extended to other homologous proteins. Thus, we analyzed nsLTPs with different allergenic potential, Mal d 3 from apple (high), Cor a 8 from hazelnut (intermediate), and Hel a 3 from sunflower seed (low),^4^, ^5^ and their interactions with 3 fatty acids: OLE, stearic (STE), and lauric (LAU) acids. We extracted natural nsLTPs and produced recombinant Mal d 3 and Cor a 8 using *Pichia pastoris*. Ligand binding of nsLTPs was assessed by ANS (1‐anilinonaphthalene‐8‐sulfonic acid) displacement assay, measuring the decrease in ANS fluorescence. IgE reactivity of nsLTPs, alone or bound to fatty acids, was tested by IgE‐ELISA using sera from subjects sensitized to peach and/or hazelnut nsLTP. For selected nLTPs/ligand complexes, molecular dynamic (MD) simulations were performed to analyze the impact of ligand binding on the protein conformation with a focus on specific IgE epitopes (details on methods and patients’ data are presented in the Supporting Information).

**Figure 1:**
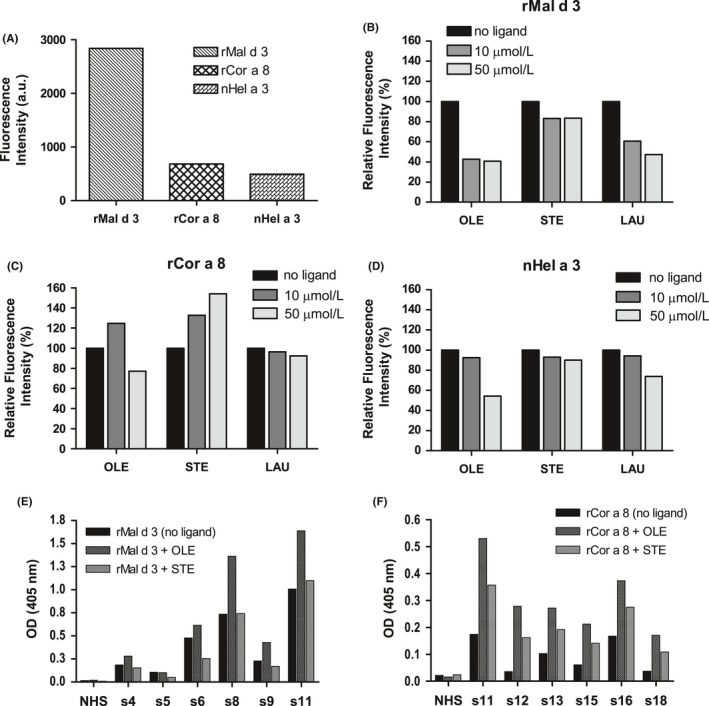
A‐D. ANS displacement assay. (A) Fluorescence of ANS in the presence of different proteins. B, C, D. ANS fluorescence changes induced by incubating purified rMal d 3 (B), rCor a 8 (C), and nHel a 3 (D) with OLE, STE, or LAU at different molar ratios (protein:ligand 1:1 = 10 μmol/L ligand or 1:5 = 50 μmol/L ligand). (E‐F) IgE‐ELISA. Effect of ligand binding on the IgE reactivity of human sera to purified recombinant proteins: (E) rMal d 3 and (F) rCor a 8 alone (no ligand) or with ligands (OLE, STE), 6 serum samples. NHS: means of 3 samples

The different natural and recombinant nsLTPs were purified and characterized (Figure S1). rMal d 3, rCor a 8, and nHel a 3 bound the ANS probe to varying extent (Figure 1A). All proteins showed the lowest preference for STE (Figure 1B‐D) with a maximum fluorescence reduction of 17% for rMal d 3/STE (50 μmol/L). LAU induced a dose‐dependent reduction of ANS binding to all proteins, reaching values at 50 μmol/L of 53%, 7%, and 26% for rMal d 3, rCor a 8, and nHel a 3, respectively. In general, OLE induced a more pronounced reduction of ANS fluorescence, equal to 59%, 23%, and 46% at 50 μmol/L for rMal d 3, rCor a 8, and nHel a 3, respectively.

Eighteen patients’ sera containing specific IgE to Pru p 3 and/or Cor a 8 (Table S1) were tested by direct IgE‐ELISA applying rMal d 3, rCor a 8, and nHel a 3 (Figure S2). We used selected sera (n = 6 for rMal d 3 and rCor a 8 each) to test if the IgE binding to the proteins is influenced by the interaction with OLE and STE, chosen as representatives of unsaturated and saturated fatty acid, respectively. Due to its very low IgE reactivity, nHel a 3 was excluded from this analysis. Preincubation of rMal d 3 with OLE significantly increased IgE‐binding (*P* < 0.05), whereas STE did not affect rMal d 3 IgE‐binding properties (Figure 1E). Regarding rCor a 8, both fatty acids induced an increase in IgE‐binding, but it was statistically significant only for OLE (*P* < 0.01; Figure 1F).

**Figure 2:**
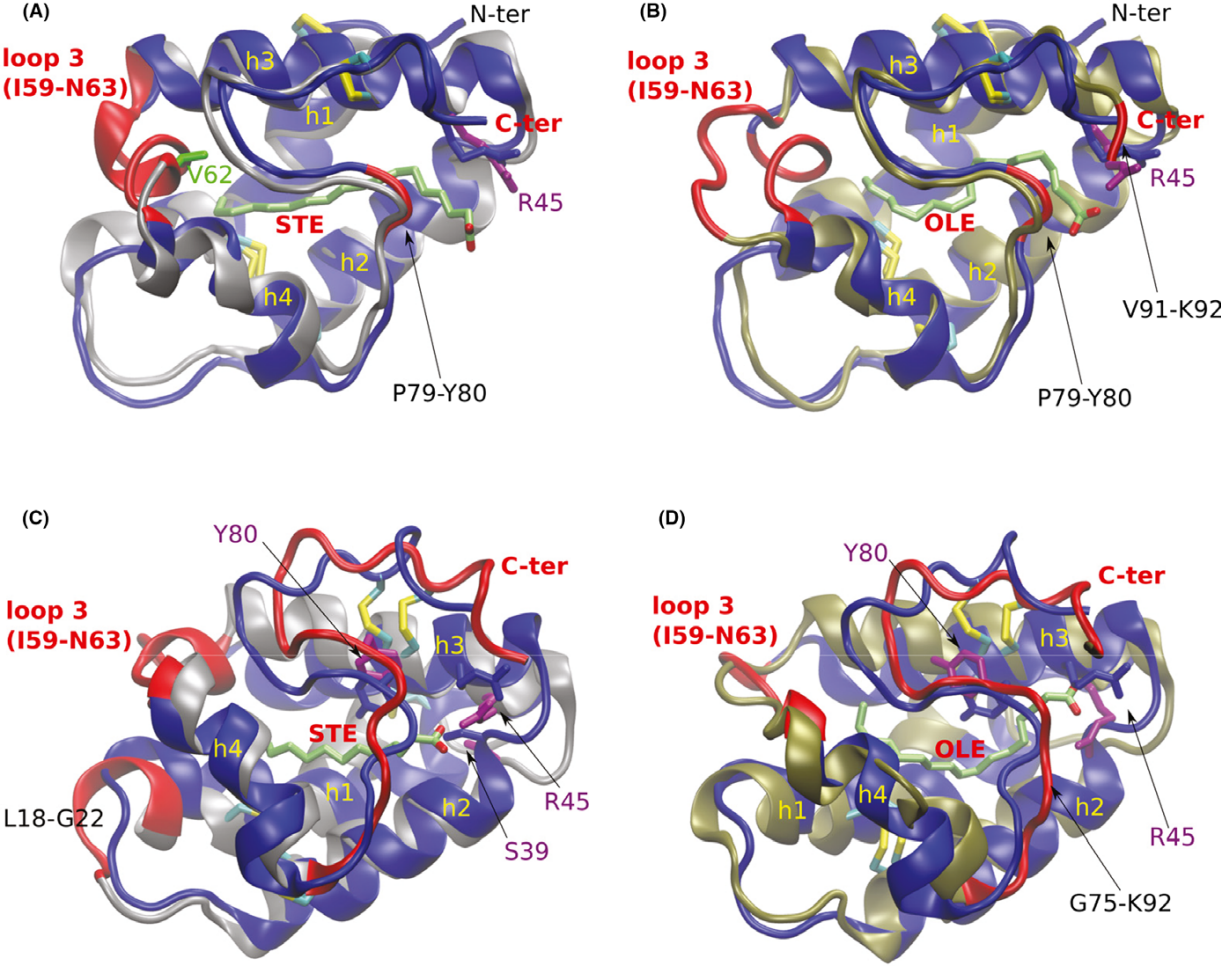
Molecular dynamic (MD) analysis. A, B. Superposed structures of apo‐Mal d 3 and Mal d 3/STE (A) and of apo‐Mal d 3 and Mal d 3/OLE (B). C, D. Superposed structures of apo‐Cor a 8 and Cor a 8/STE (C) and of apo‐Cor a 8 and of Cor a 8/OLE (D). Apo forms of the allergens are in blue and the ligand‐bound forms in grey (STE) and gold (OLE). The regions in red are those affected by ligand binding; important residues are highlighted. *Note*: The Mal d 3 model used for computations has one additional amino acid, Ala, at the N‐terminus and, hence, the indexes of protein residues discussed for the 3D model are by 1 larger with respect to the sequence reported in Figure S1

We performed computational calculations, detecting some differences in the cavity size of the apo nsLTPs: Mal d 3 had a larger pocket size (124 Å^3^) compared to both Cor a 8 and Hel a 3 (106 Å^3^ and 111 Å^3^, respectively). MD simulations were performed on Mal d 3 and Cor a 8 in complexes with either OLE or STE. For both proteins, the inclusion of ligands significantly increased the cavity volume, affecting the protein conformation. As shown in Figure 2, both allergens underwent conformational changes upon OLE binding, especially displacing loop 3 (I59 to N63) of Mal d 3, which moved toward the allergen surface, and affecting the C‐terminal region in both allergens, which is consistent with our previous findings.^3^ Notably, this C‐terminal region has been identified in Pru p 3 as a major IgE epitope,^6^ thus explaining the increased IgE reactivity observed for the nsLTP/OLE complexes. Interestingly, we observed similar conformational changes for the Cor a 8/STE complex, although to a minor extent, which was paralleled by a slight, albeit not statistically significant increase in IgE reactivity. On the contrary, binding of STE to Mal d 3 did not induce any structural change in the allergen, but rather increased its stability, yet without changing the IgE‐binding properties.

Considering differences in the allergenic potential of homologous proteins, we should take into account *“the necessity of other matrix component to induce allergenic responses or differences and particularities in the epitope composition*.”^7^ Here, we focused on both food matrix and epitopes. With regard to the latter, the epitope sequences identified in Pru p 3 are well conserved in Mal d 3, but less in the other two nsLTPs, especially in Hel a 3 (Figure S1A)^6^. This could explain its very low IgE‐binding, as in most cases, Pru p 3 is the primary sensitizer.

Regarding the food matrix, we focused on how the interaction with fatty acids, commonly present in the food sources,^8^ could affect the allergenic properties of nsLTPs. Consistently with our previous observation for Pru p 3,^3^ all nsLTPs had the highest preference for OLE, whose binding induced major structural changes that affected IgE epitope orientation, and thus, their recognition by IgE, in both, Mal d 3 and Cor a 8.

Recently, the direct immunomodulatory effect of food‐derived lipids has gained increasing interest. Tordesillas et al showed the adjuvant activity of the natural ligand of Pru p 3 in the allergen sensitization.^9^ Summarizing the recent findings, including our data, it still remains to be fully elucidated whether lipids alone or in complexes can activate key players of both, the innate and adaptive immune system within an allergic response.

In conclusion, this multidisciplinary study analyzed for the first time the ligand‐binding capacities of Mal d 3, Cor a 8, and Hel a 3 and confirmed that these individual homologous allergens display different, higher and lower, IgE‐binding activities, due to differences in their epitope structure.

Furthermore, the volume and structural properties of the ligand‐binding cavity of individual nsLTPs are critical for their ligand‐binding activities. Upon ligand binding, the accessibility of these epitopes changed. Taken together our data provide molecular evidence how ligand binding to nsLTP affects IgE‐binding activity.
