# Supplementary material for: Distinct Lipid Transfer Proteins display different IgE‐binding activities that are affected by fatty acid binding
Source: Allergy. 2018 Dec 16;74(4):827–31. doi: 10.1111/all.13682 (PMC6491988; doi:10.1111/all.13682)
Supplement: Supplementary file 1 [file ALL-74-827-s001.pdf]

SUPPORTING INFORMATION

Distinct Lipid Transfer Proteins display different IgE-binding activities that are affected by fatty acid binding

Roberta Aina[1], Pawel Dubiela[1], Sabine Geiselhart[1], Merima Bublin[1], Maurizio Bruschi[2], Christian Radauer[1], Christoph Nagl[1], Piotr Humeniuk[1], Riccardo Asero[3], Charlotte Gotthard Mortz[4], Christine Hafner[5,6], Karin Hoffmann-Sommergruber[1*§] and Tomasz Borowski[7*].

[*]*shared last authorship*

[§]*corresponding author*

[1]Department of Pathophysiology and Allergy Research, Medical University of Vienna, Vienna, Austria.

[2]Department of Earth and Environmental Science, University of Milano Bicocca, Milan, Italy.

[3]Ambulatorio di Allergologia, Clinica San Carlo, Paderno Dugnano (MI), Italy.

[4]Department of Dermatology and Allergy Center, Odense Research Center for Anaphylaxis (ORCA), Odense University Hospital, Odense, Denmark.

[5]Department of Dermatology, University Hospital St. Poelten, Karl Landsteiner University of Health Sciences, St. Pölten, Austria.

[6]Karl Landsteiner Institute of Dermatological Research, Karl Landsteiner Gesellschaft, St. Pölten, Austria.

[7]Jerzy Haber Institute of Catalysis and Surface Chemistry, Polish Academy of Sciences, Krakow, Poland.

Correspondence

Karin Hoffmann-Sommergruber

Department of Pathophysiology and Allergy Research, Medical University of Vienna, Währinger Gürtel 18-20, A-1090 Vienna, Austria.

E-mail: karin.hoffmann@muv.ac.at

SHORT TITLE

Ligand binding of nsLTPs affects IgE recognition

METHODS

Patients

Sera from subjects (n=18) sensitized to peach and/or hazelnut nsLTP and from non-atopic donors (n=3; NHS) were collected at the Clinica San Carlo (Paderno Dugnano, Milan, Italy), at the Odense Research Center for Anaphylaxis (Odense University Hospital, Odense, Denmark), at the University Hospital St. Pölten (Austria) and at the Medical University of Vienna (Austria). Diagnosis of peach or hazelnut allergy was based on a convincing case history and positive skin prick test to the offending food in 15 subjects, 3 were sensitized without any clinical history of food allergy but were birch pollen allergic. Pru p 3- and/or Cor a 8- specific IgE levels were evaluated by means of Immuno-CAP (ThermoFisher Scientific). The use of serum samples for this study was approved by the local Ethics Committees, performed according to Declaration of Helsinki and informed written consent was obtained from all participants.

Materials

If not stated otherwise, chemicals were purchased from Sigma-Aldrich (Saint Louis, MS, USA).

Purification of natural and recombinant nsLTPs

Natural Mal d 3 was extracted from apple (*Malus domestica* cv. Golden Delicious) peel and purified by cation-exchange chromatography, according to Dubiela et al.[3] Natural Cor a 8 was extracted from shelled, raw hazelnuts (*Corylus avellana*, Beach Flower Back Mix, Turkey) as previously reported,[S1] whereas nHel a 3 was purified from sunflower (*Helianthus annuus*) seeds as described by Burnett et al.[S2] Recombinant Mal d 3 and rCor a 8 were produced in the yeast *Pichia pastoris* using the pPICZαA vector (ThermoFisher Scientific, Waltham, MA, USA) and purified following our established protocols.[3]

Protein characterization

Intact masses of purified rMal d 3 and rCor a 8 were determined by MALDI-TOF-MS (Microflex, Bruker Daltonics, Bremen, Germany) and their secondary structures were analyzed by CD spectroscopy. The N-terminal sequences of all purified natural and recombinant proteins were determined using an Applied Biosystems Procise 491 sequencer (Applied Biosystems, Foster City, CA, USA). All the protocols are reported in Dubiela et al.[3] Purified nsLTPs were analyzed by 15% SDS-PAGE and by immunoblotting with rabbit antiserum against natural Cor a 8.

ANS (1-anilinonaphthalene-8-sulfonic acid) displacement assay

The ANS displacement assay was performed to detect protein-ligand binding as previously reported.[3] The unsaturated fatty acid, OLE (C18:1), and the two saturated ones, STE (C18:0) and LAU (C12:0), were assessed for their abilities to compete with the fluorescent probe ANS for the binding to purified nsLTPs. rMal d 3, rCor a 8 and nHel a 3 (10 µM) were incubated with each ligand at molar ratios of 1:1 and 1:5 (protein:ligand). Ligand binding was analyzed by adding 10 µM ANS and measuring the ANS fluorescence at 456 nm, after excitation at 350 nm. All samples were analyzed in triplicates. Purified proteins without ligands and ligands alone were used as reference and negative controls, respectively.

IgE ELISA

The IgE-binding capacities of purified nsLTPs were tested by standard direct IgE ELISA as described previously.[S3] To compare the IgE reactivities to apo- and ligand-bound forms of rMal d 3 and rCor a 8, allergens were covalently bound to Thermo Scientific™ Nunc™ Immobilizer™ Amino surface plates at native conditions in the presence or absence of ligands, as reported by Dubiela et al.[3] Statistical analyses were performed with GraphPad Prism (GraphPad Software, La Jolla, CA, USA), using the Friedman test followed by Dunn's test.

Computational calculations and molecular dynamics analysis

The structure of Mal d 3 was modelled on Pru p 3 (PDB:2ALG) using the SWISS-MODEL server,[S4] as previously reported,[S5] whereas molecular modelling of Hel a 3 was carried out by using I-TASSER server (https://zhanglab.ccmb.med.umich.edu/I-TASSER/).[S6] Initial coordinates of Cor a 8 were taken from the crystal structure (PDB: 4XUW).[S2] We used the CASTp server (http://sts.bioe.uic.edu/castp/index.php)[S7] to define the size of the cavities of the proteins. Molecular dynamics (MD) simulations were performed on Mal d 3 and Cor a 8, using the AMBER force field ff03.r1 for the proteins.[S8] Molecular force fields of OLE and STE anions were described with the general AMBER force field GAFF.[S9] System preparation and simulation setup have been performed as previously described.[3]

RESULTS

Protein characterization

Results of the multiple sequence alignment of the studied nsLTPs are shown in Figures S1A,B. MS analysis of purified rMal d 3 and rCor a 8 confirmed their identities, providing 9,552 Da for rMal d 3 and 10,160 Da for rCor a 8 (Figures S1C,E). These values correspond to the calculated masses of mature Mal d 3 (9,552 Da) and Cor a 8 (10,145 Da) with 4 (EAEF) or 6 (EAEAEF) additional residues, respectively, which result from the cleavage of the α-factor signal peptide, as confirmed by N-terminal sequencing. CD analysis showed a good agreement of the secondary structures of the recombinant and the corresponding natural proteins (Figures S1D,F), providing evidence of a typical α-helical structure. In SDS-PAGE, all nsLTPs migrated between 11 and 13 kDa with no additional protein bands (Figure S1G). All purified proteins were recognized by a rabbit anti-nsLTP antiserum (Figure S1H). After having verified the equivalence of physicochemical properties of purified nMal d 3 and rMal d 3, as well as nCor a 8 and rCor a 8, the recombinant proteins and nHel a 3 were used throughout all further experiments.

Dynamic behaviour of the allergen-ligand complexes

From comparison of the starting structure of apo Cor a 8 and the representative snapshot from its MD trajectory it follows that larger displacements took place for the terminal regions and for the loop connecting α-helices 3 and 4 (loop 3) (data not shown). Computed per-residue B-factor indicate that these regions, along with loop 1, were the most flexible parts of the protein. Comparison of per-residue B-factor (Figure S3), RMSD (Figures S4), 2D-RMSD and Radius of Gyration plots (data not shown) for apo Cor a 8 and its complexes with OLE or STE indicates that the structure was substantially stabilized by ligand binding. This increase in rigidity was accompanied by several structural changes. Binding of STE (Figure 2C) affected the regions corresponding

to loop 1 (L18-G22; RMSD of 4.5 Å); loop 3 (I59-N63; RMSD of 4.1 Å) and the C-terminal tail (G75-K92; RMSD of 4.2 Å). In the case of OLE, a large conformational change of the C-terminal tail was observed (RMSD 4.7 Å; Figure 2D), which mainly affected the last amino acids. Interestingly, STE did not show any significant interactions with this C-terminal fragment, as inferred from the native contact analysis, yet its presence reduced flexibility of this region, as the computed B-factors indicate. In contrast, OLE interacted slightly with these residues and increased their flexibility. The effect of the ligands on mobility of the C-terminus may be indirect. We observed that in Cor a 8, and also Mal d 3, a conserved Arg residue (R45), which belongs to α-helix 3, is involved in a salt bridge with the carboxylic group of the C-terminus, which might indicate this residue has an important structural role. Importantly, R45 was also involved in a strong interaction with the carboxylic group of OLE and to a somewhat lower extent with the carboxylic group of STE. In the Cor a 8/STE complex we also observed that the hydroxyl group of Y80 interacts with the carboxylic group of the ligand.

Like in the case of Cor a 8, from comparison of the starting structure and the representative snapshot from MD trajectory of apo Mal d 3 it follows that larger displacements took place for the terminal regions and loop 3. However, the two ligands had opposite effects on the dynamic behavior of this protein. Hence, the complex Mal d 3/OLE was substantially more flexible than the apo form, whereas binding of STE had a neutral / slightly stabilizing effect, as inferred from per-residue B-factor (Figure S3), RMSD (Figure S4), 2D-RMSD and Radius of Gyration plots (data not shown). Like in the case of Cor a 8/OLE, in the Mal d 3/OLE complex the structure of the C-terminus was affected (V91-K92, RMSD = 3.5 Å), and similarly OLE made this fragment more flexible, whereas STE more rigid, through direct and indirect (R45) interactions. Binding of STE to Mal d 3 also induced a moderate conformational change in loop 3 (residues I59 to N63, backbone RMSD 3.5 Å), which became slightly less mobile; Native contacts analysis revealed V62 played a major role in the contact with the ligand (data not shown). Binding of OLE to Mal d 3 (Figure 2B) also affected loop 3, which moved towards the surface of the molecule (RMSD of 6.2 Å). Notably, opposite to the effect of STE, this region was even more mobile than in apo-Mal d 3. This may be caused by the different structure of OLE compared to STE, preventing this region from interacting with the ligand (Figure 2B)

REFERENCES

S1 Offermann LR, Bublin M, Perdue ML et al. Structural and Functional Characterization of the Hazelnut Allergen Cor a 8. *J Agric Food Chem.* 2015;63:9150-9158.

S2 Burnett GR, Rigby NM, Mills EN et al. Characterization of the emulsification properties of 2S albumins from sunflower seed. *J Colloid Interface Sci.* 2002;247:177-185.

S3 Pfeifer S, Bublin M, Dubiela P et al. Cor a 14, the allergenic 2S albumin from hazelnut, is highly thermostable and resistant to gastrointestinal digestion. *Mol Nutr Food Res.* 2015;59:2077-2086.

S4 Biasini M, Bienert S, Waterhouse A et al. SWISS-MODEL: modelling protein tertiary and quaternary structure using evolutionary information. *Nucleic Acids Research.* 2014;42(Web Server issue):W252-W258. doi: 10.1093/nar/gku340.

S5 Alessandri S, Sancho A, Vieths S et al. High-throughput NMR assessment of the tertiary structure of food allergens. *PLoS One.* 2012;7:e39785.

S6 Yang J, Yan R, Roy A, Xu D, Poisson J, Zhang Y. The I-TASSER Suite: protein structure and function prediction. *Nat Methods.* 2015;12:7-8.

S7 Dundas J, Ouyang Z, Tseng J, Binkowski A, Turpaz Y, Liang J. CASTp: computed atlas of surface topography of proteins with structural and topographical mapping of functionally annotated residues. *Nucleic Acids Res* 2006;34(Web Server issue):W116- W118.

S8 Duan Y, Wu C, Chowdhury S et al. A point-charge force field for molecular mechanics simulations of proteins based on condensed-phase quantum mechanical calculations. *J Comput Chem.* 2003;24:1999-2012.

S9 Wang J, Wolf RM, Caldwell JW, Kollman PA, Case DA. Development and testing of a general amber force field. *J Comput Chem* 2004;25:1157-1174. Erratum in: *J Comput Chem.* 2005;26:114.

S10 Sievers F, Wilm A, Dineen D et al. Fast, scalable generation of high-quality protein multiple sequence alignments using Clustal Omega. *Mol Syst Biol.* 2011;7:539.

S11 Goujon M, McWilliam H, Li W et al. A new bioinformatics analysis tools framework at EMBL-EBI. *Nucleic Acids Res* 2010;38(Web Server issue):W695-W699. doi: 10.1093/nar/gkq313

S12 García-Casado G, Pacios LF, Díaz-Perales A et al. Identification of IgE-binding epitopes of the major peach allergen Pru p 3. *J Allergy Clin Immunol.* 2003;112:599-605.

SUPPLEMENTARY FIGURES AND TABLES

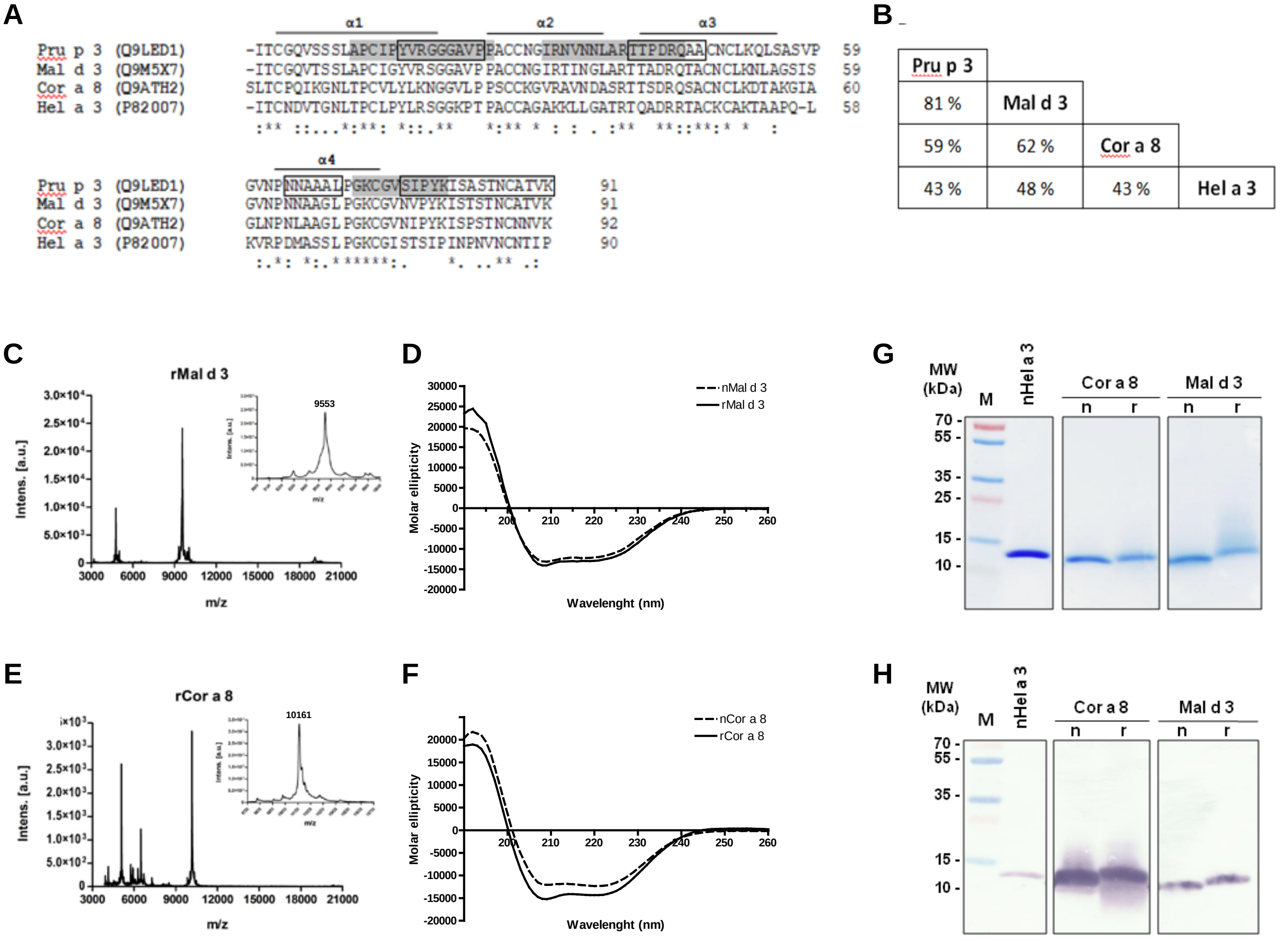

Figure S1

A, B. Multiple sequence alignment (Clustal Omega v1.2.4; http://www.ebi.ac.uk/Tools/msa/clustalo/)[S10,S11] and percent identity matrix of nsLTP sequences from peach (Pru p 3.0102, as reference), apple (Mal d 3.0101), hazelnut (Cor a 8.0101), and sunflower (Hel a 3.0101). Amino acid sequences corresponding to the linear IgE epitopes identified in Pru p 3 by Borges et al.[6] and Garcia-Casado et al.[S12] are enclosed in boxes and highlighted in grey, respectively. The sequences corresponding to the 4 α-helices (α1 to α4) are also indicated. (*) = positions which have a single, fully conserved residue; (:) = conservative position (amino acid with the same properties) (.) = semi-conservative position (amino acid with weakly similar properties). **C, E.** MALDI-TOF MS analysis of rMal d 3 and rCor a 8. **D, F**. Far-UV CD spectra of rMal d 3 and rCor a 8, compared with the natural forms. Spectra represent the average of three accumulations. **G.** Representative Coomassie stained 15% SDS-PAGE of the purified natural (n) and recombinant (r) proteins. Protein molecular weight was estimated by using the PageRuler™ Plus Prestained Protein Ladder (M; Thermo Scientific). **H.** Immunoblots with rabbit anti-nsLTP antiserum (colorimetric detection).

Table S1: Clinical data of the patient population

Patient	Country	IgE (kU/L) specific to		SPT to plant food	Symptoms
		Pru p 3	Cor a 8		
1	I	1.27	-	P	*OAS:* P, A
2	I	1.21	-	P	*OAS*: P, Ch, Ap juice, K
3	I	0.54	-	P	*OAS*: P, A, K
4	I	2.29	-	P	*OAS*: T, Or, Au, Pa
5	I	4.78	-	P	*OAS*: P, P juice, Ap, Al, Pn, Wn, Hn, Me
6	I	6.99	-	P	*Urticaria*:P, Ap<br>*OAS*: Me, Wm, St
7	I	5.50	-	P	*OAS*: several fruits
8	I	48.10	-	P, Hn, Wn, Pn, T	*Urticaria, angioedema*: T, nuts
9	I	11.80	-	P	*Urticaria, angioedema*
10	I	4.91	-	P	*OAS*: T
11	A	-	13.40	-	*Urticaria, angioedema*: Hn, Wn<br>*Dyspnea*:Hn, Wn<br>*GI*: T, Ce, K, Ba
12	D	1.90	1.00	Hn, Wh, Pn, So, Bn, B, O, R, S, Su, Ps, Bu	
13	D	2.30	1.10	Hn, Pn, So, Bn, B, R, Bu	
14	D	4.40	14.00	Hn, Wh, Pn, So, Wn, Bu	
15	A	-	1.65	Ry	*Anaphylaxis*: Hn, Pn
16	A	-	0.76	Hn, Pn, So, T, R	*Urticaria:* Hn, Pn, Wn, Mu<br>*Asthma:* Hn, Pn, Wn, Mu
17	A		0.27	-	*Urticaria:* Hn, Pn, Wn, Mu<br>*Asthma:* Hn, Pn, Wn, Mu
18	A	-	0.98	Hn, Pn, T, Wh, C	*Urticaria, local swelling:* Hn, Pn<br>*Diarrhea, vomiting:* Pn<br>*Anaphylaxis*: Pn

I = Italy; A = Austria; D = Denmark. OAS, oral allergy syndrome; *GI*, gastrointestinal tract.
A, apple; Al, almond; Ap, apricot; Au, aubergine; B, barley; Ba, banana; Bn, brazil nut; Bu, buckwheat; C, cashew; Ce, celery; Ch, cherry; Hn, hazelnut; K, kiwi; Me, melon; Mu, mustard; O, oat; Or, orange; P, peach; Pa, pineapple; Pn, peanut; Ps, poppy seed; R, rape; Ry, rye; S, sesame; So, soy; St, strawberry; Su, sunflower seed; T, tomato; Wh, wheat; Wm, watermelon; Wn, walnut.
-, not determined.

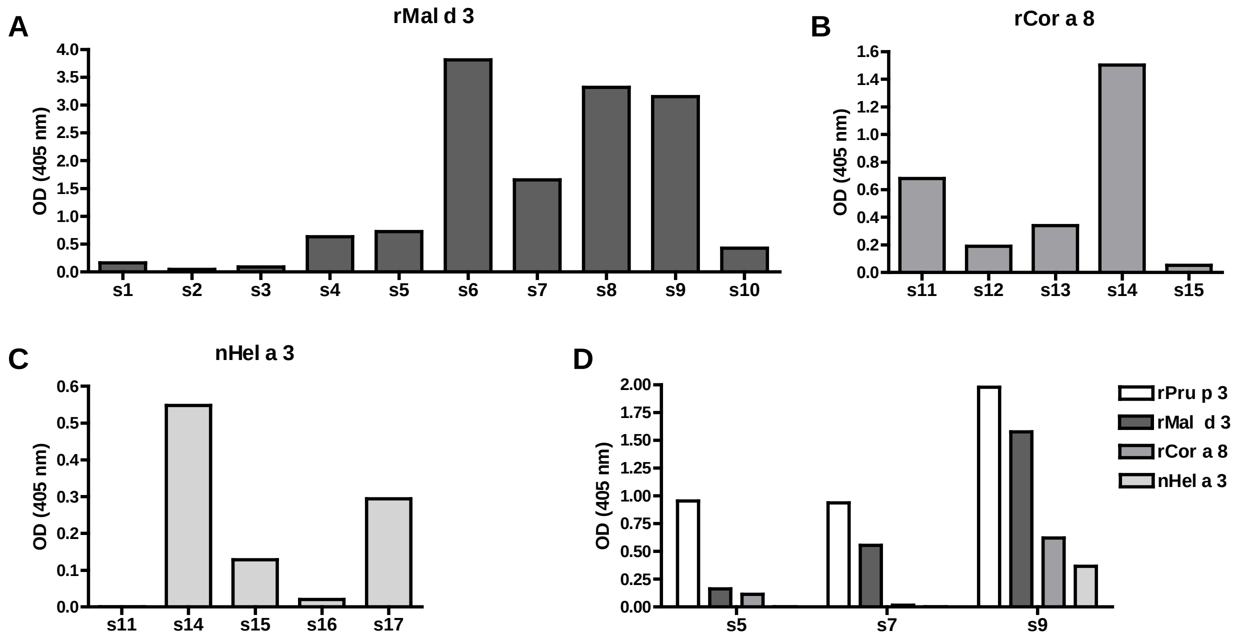

Figure S2

Representative IgE ELISA with different nsLTPs: rMal d 3 (**A**); rCor a 8 (**B**); nHel a 3 (**C**); comparison of IgE reactivity of the nsLTPs with selected human sera **(D).** rPru p 3 was used as a reference/positive control. OD values were counted positive if they exceeded the mean OD of the negative controls (3 NHS) by more than three standard deviations.

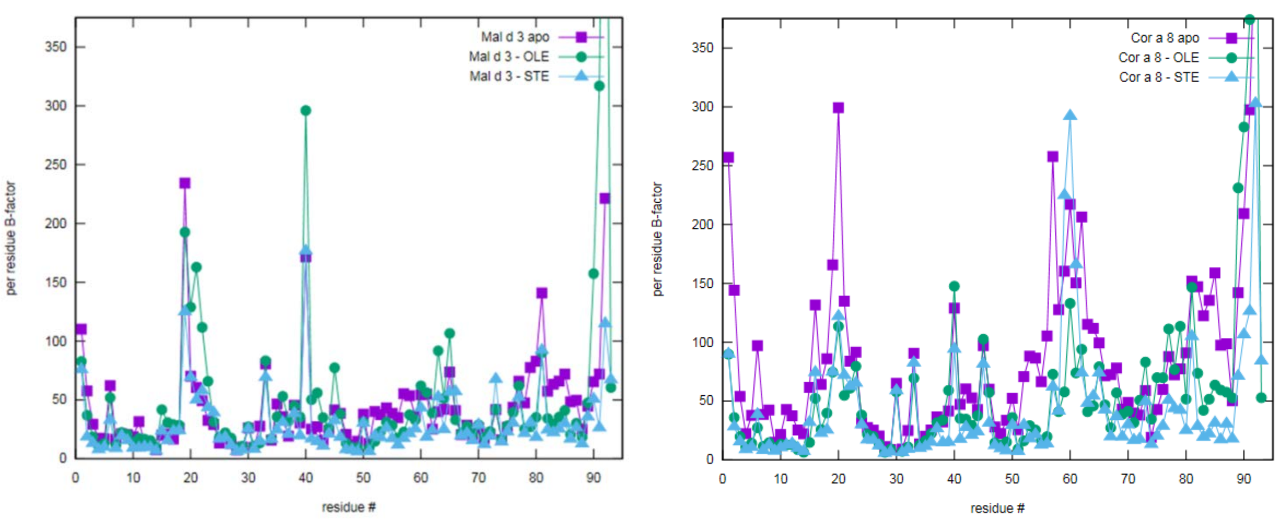

<

<

Figure S3

Per-residue B-factor plots of apo- and ligand-bound form of Mal d 3 (left) and Cor a 8 (right).

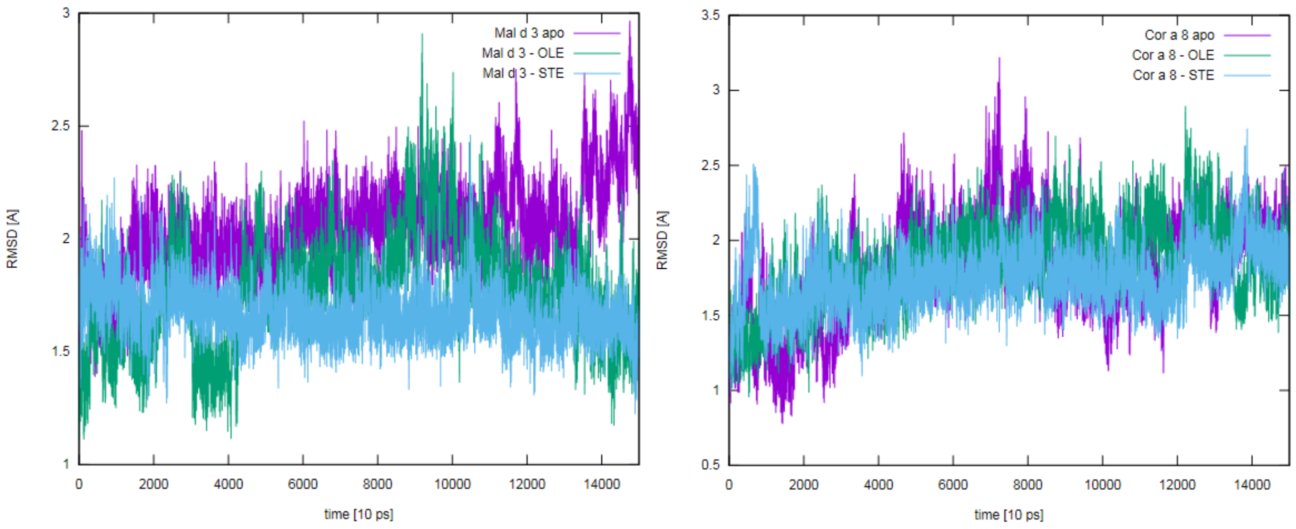

Figure S4

RMSD plots of apo- and ligand-bound form of Mal d 3 (left) and Cor a 8 (right).
